# Mortality among persons experiencing musculoskeletal pain: a prospective study among Danish men and women

**DOI:** 10.1186/s12891-020-03620-8

**Published:** 2020-10-08

**Authors:** Teresa Holmberg, Michael Davidsen, Lau Caspar Thygesen, Mikala Josefine Krøll, Janne Schurmann Tolstrup

**Affiliations:** 1 National Institute of Public Health, University of Southern Denmark, Studiestræde 6, DK-1455 Copenhagen, Denmark; 2 Zealand University Hospital, Region Zealand, Denmark

**Keywords:** Mortality, Musculoskeletal pain, Prospective, Pain intensity, Population-based

## Abstract

**Background:**

Musculoskeletal (MSK) pain affects many people worldwide and has a great impact on general health and quality of life. However, the relationship between MSK pain and mortality is not clear. This study aimed to investigate all-cause and cause-specific mortality in relation to self-reported MSK pain within the last 14 days, including spread of pain and pain intensity.

**Methods:**

This prospective cohort study included a representative cohort of 4806 men and women aged 16+ years, who participated in a Danish MSK survey 1990–1991. The survey comprised questions on MSK pain, including spread of pain and pain intensity. These data were linked with the Danish Register of Causes of Death to obtain information on cause of death. Mean follow-up was 19.1 years. Cox regression analyses were performed with adjustment for potential confounders.

**Results:**

In the study population (mean age 44.5 years; 47.9% men), 41.0% had experienced MSK pain within the last 14 days and 1372 persons died during follow-up. For both sexes, increased all-cause mortality with higher spread and intensity of MSK pain was observed; a high risk was observed especially for men with strong pain (HR = 1.66; 95% CI:1.09–2.53) and women with widespread pain (HR = 1.49; 95% CI:1.16–1.92). MSK pain within last 14 days yielded c-statistics of 0.544 and 0.887 with age added. Moreover, persons with strong MSK pain had an increased cardiovascular mortality, persons with moderate pain and pain in two areas had an increased risk of cancer mortality, and persons with widespread pain had an increased risk of respiratory mortality.

**Conclusions:**

Overall, persons experiencing MSK pain had a higher risk of mortality. The increased mortality was not accounted for by potential confounders. However, when evaluating these results, it is important to take the possibility of unmeasured confounders into account as we had no information on e.g. BMI etc.

**Significance:**

The present study provides new insights into the long-term consequences of MSK pain. However, the discriminatory accuracy of MSK pain was low, which indicates that this information cannot stand alone when predicting mortality risk.

## Background

Musculoskeletal (MSK) conditions, including MSK diseases and pain, are a major public health burden globally [1, 2]. According to the *Global Burden of Disease Study 2017,* the leading cause of years lived with disability (YLD) globally is low back pain, with neck pain and other MSK conditions among the top fifteen causes of YLD [1]. MSK conditions combined account for 21% of the total global YLD and approximately 7% of all years of healthy life lost [2]. The global prevalence of MSK pain is estimated to be approximately 30% [3], and in Denmark, around half of the population has experienced some kind of MSK pain within a given two-week period [4].

Musculoskeletal conditions are prevalent across all age and socioeconomic status groups and, due to the longer life exp*e*ctancy and changes in lifestyle, are estimated to increase worldwide [5, 6]. Even though it is well established that MSK pain is a major social burden with an impact on general health and quality of life [2, 5, 7, 8], the relationship between MSK pain and mortality is still uncertain. Some existing studies have reported positive associations between MSK pain and increased mortality [9–13], whereas other of the studies surveyed found no significant association, in particular after controlling for various demographic and behavioural factors [14–21].

Overall, there is a need for studies exploring the relationship between MSK pain and mortality, and studies accounting for other explanatory factors have been requested [14, 22]. Therefore, the aim of the current study was to investigate all-cause and cause-specific mortality in relation to self-reported MSK pain within the last 14 days, including pain intensity and spread, using a Danish representative MSK cohort with nearly 25 years of follow-up in national registers. Our hypothesis is that MSK pain increases mortality. This present study is important for various reasons. First, we want to explore whether an association remains significant even after accounting for other important explanatory factors such as educational level and comorbidity. Moreover, analyses will include considerations of potential effect modifications, and include pain intensity, which has not been fully investigated.

## Method

### Study-design and population

Ever since 1987, the National Institute of Public Health in Denmark has conducted nationally representative surveys of the adult Danish population aged 16 years or older. In this prospective cohort study, we used the survey from 1990 to 1991, where 5986 persons were chosen at random from the adult Danish population and invited to answer an interview-based face-to-face questionnaire with specific focus on musculoskeletal conditions (42 out of 97 items in the questionnaire were related to musculoskeletal conditions, e.g. location, degree and duration of pain, treatment and prevention strategies). The data collection was conducted in three sessions: in September 1990, February 1991 and May 1991.

### Variables

#### MSK pain within the last 14 days

Respondents with musculoskeletal pain were identified through the use of a questionnaire that included questions on MSK pain within the last 14 days. In the current study the following MSK variables were used:

- MSK pain within the last 14 days (yes/no): Have you during the past 2 weeks had trouble (pain or discomfort) in any of these locations: neck, shoulders, upper back, elbows, low back, hands/wrist, hip, knee, or feet/ankles. To aid the respondent in answering this question, the interviewer showed a diagram with the anatomical areas clearly shaded and labelled. The diagram was from the ‘Standardised Nordic questionnaires for the analysis of musculoskeletal symptoms’ [23]. Each person could respond ‘yes’ or ‘no’ to any of the complaints, i.e., more than one confirmatory answer was allowed. Persons with MSK pain in one or more locations were categorised with MSK pain within the last 14 days.
- Pain in different locations was categorised into three different regions: upper body (elbows, shoulders, hands), lower body (hips, knees, feet/ankles), and trunk (neck, chest, upper- and lower back) based on the answers to the above-mentioned questions, and thereafter categorized into a variable holding information on spread of pain (no pain; 1 region; 2 regions; 3 regions (widespread)), a modified version of the American College of Rheumatology’s 1990 definition [24] and inspired by Overland and colleagues [25].
- Pain intensity was used as an overall measure related to the location where respondents experienced most pain/discomfort and was measured on a pain scale (visual analog scale (VAS) from ‘0’ (no pain) to ‘100’ (unbearable pain)) included both as a continuous variable and grouped into four categories as formulated by Hawker and colleagues: (0–4 (no pain); 5–44 (mild pain); 45–74 (moderate pain); 75–100 (severe pain) [26]#*.* Persons reporting no MSK pain within the last 14 days were categorised into the no-pain group.

#### Cause of death and register follow-up

All persons residing in Denmark are assigned a unique personal identification number (CPR number). The CPR number is used consistently across all national Danish registers and may therefore be used to link data from surveys with registers at an individual level [27]. In this study, data from the 1990–1991 survey were linked to information on date and cause of death from the Danish Register of Causes of Death (RCD). The RCD contains data on all deaths registered since 1875, and since 1970 it includes electronic individual records on, among other things, cause of death, place, date, and manner of death [28]. Cause of death was classified using the International Classification of Diseases version 8 (ICD-8) from 1990 to 1993 and version 10 (ICD-10) from 1994 and onwards. Causes of death were categorized into the following disease groups: cardiovascular disease (ICD-8: 390.0–458.0; ICD-10:I00-I99), cancer (ICD-8: 140.0–209.0; ICD-10: C00-C99), respiratory diseases (ICD-8: 460.0–474.0, 480.0–486.0, 490.0–493.0, 500.0–519.0; ICD-10: J00-J99), psychological diseases and Alzheimer’s/dementia (ICD-8: 290.0–290.9; ICD-10: F01, F03–99, R54), gastrointestinal diseases (ICD-8: 520.0–577.0; ICD-10: K00-K99) and other causes. Information on emigration was extracted from the Civil Registration System (CRS).

#### Baseline confounders

Based on various studies on the epidemiology of MSK pain, a number of potential confounders that could affect the relationship between MSK pain and mortality were selected: marital status; physical activity; social support; stress; smoking; educational level; age, and comorbidity [3, 7, 29, 30]. For example, it is known that the prevalence of MSK pain is higher in older persons, and age is a risk factor for all-cause mortality. Selected confounders were measured at baseline. Marital status was grouped into four categories: married or cohabitant, widow/widower, separated or divorced and unmarried. Physical activity comprised four categories: heavy exercise and competitive sports several times a week (vigorous or moderate physical activity); walking, cycling, or other light exercise at least 4 h a week (light physical activity); reading, watching TV, or other sedentary activity (sedentary physical activity). Level of social support was addressed with a question on how often they meet up with friends or acquaintances: daily or almost daily; 1–2 times a week or a month; less often or never. Stress in daily life was categorized (often, from time to time, never or almost never, and don’t know). Finally, the respondents’ smoking behaviour was assessed in the second and third of the baseline data collection waves; the question on smoking was not included in the first wave, which is why we only have this information on 3198 of the respondents (*n* = 3198). Smoking behaviour was categorized into daily smokers, occasional smokers, former smokers and non-smokers.

The respondents’ highest education level at baseline was retrieved from the Population Education Register. Educational level was classified according to the International Standard Classification of Education (ISCED) combining (ongoing or completed) school and vocational education; < 10, 10, 11–12, 13–14, 15+ years. Due to a relatively high proportion of missing data on education (15.2%), missing was classified into a category of its own. The respondent’s age and sex at baseline were retrieved from CRS. Finally, the Charlson comorbidity index that measures burden of disease was used to classify comorbid conditions among the respondents [31]. To calculate the baseline Charlson score we used information from the National Patient Register. The register includes discharge diagnoses of hospitalised patients indicating the main medical reason for diagnostic procedures or treatment. The information was obtained in the period 1982–1991 using ICD-8 codes [32]. The Charlson score was categorized into three groups: no comorbidity (0), low comorbidity (1) and medium/high (>=2).

#### Statistics

Individuals were included in the study from date of interview. End date of follow-up was 31 December 2013, date of death or date of emigration, whichever came first. Results are presented as mean and standard error (SE), frequencies or hazard ratio (HR) with 95% confidence intervals (95% CI). For comparison between groups, the Chi^2^ test was used to evaluate categorical variables and a t-test was used to evaluate continuous variables. The cumulative incidence of all-cause mortality was calculated using Kaplan-Meier survival analysis. The discriminatory accuracy is reported using the Harrell C-index. Moreover, all-cause and cause-specific mortality rates were calculated, and Cox regression models were used to adjust for confounders. In analyses of all-cause mortality, three models were applied (1: age-adjusted; 2: adjusted for: age, marital status, social support, educational level, and 3: adjusted for: age, marital status, social support, educational level, physical activity, daily stress, comorbidity) as it could be argued that some variables could act as both confounders and/or mediators, e.g. physical activity.

Only relevant causes of death were included in the analyses; other causes were considered censored at death. Age was used as the underlying time variable in the Cox regression models, treating age at interview as the time of delayed entry, thereby adjusting for age. This is the preferable method when age is a stronger determinant of the outcome than follow-up time since study entry [33]. In order to investigate the relationship between pain intensity as a continuous variable and mortality, linearity was tested by applying age^2^ and age^3^ in the Cox regression models, and cubic splines were applied (three knots set at 25, 50 and 75 on the pain-scale).

Due to general variations in the prevalence of musculoskeletal pain and in mortality related to sex, the main analysis was stratified by sex. Likewise, we tested for multiplicative effect modification between MSK pain and education as well as MSK pain and comorbidity. Moreover, sensitivity analyses were performed in the group of informants who answered the question on smoking to determine if there were any differences in results when taking smoking behaviour into account. Finally, sensitivity analyses delaying start of follow-up with 2 years were performed to minimize the effect of pre-existing disease causing pain and death in the first period after baseline.

STATA v. 15.0 (StataCorp LP, TX, USA) statistical software package was used for statistical analysis. All Cox regression analyses were tested for the proportional hazard assumption using the STATA stphtest command, checking the proportional-hazards assumption on the basis of Schoenfeld residuals after fitting a model with stcox; in which the proportional hazard assumption was fulfilled.

## Results

**Fig. 1:**
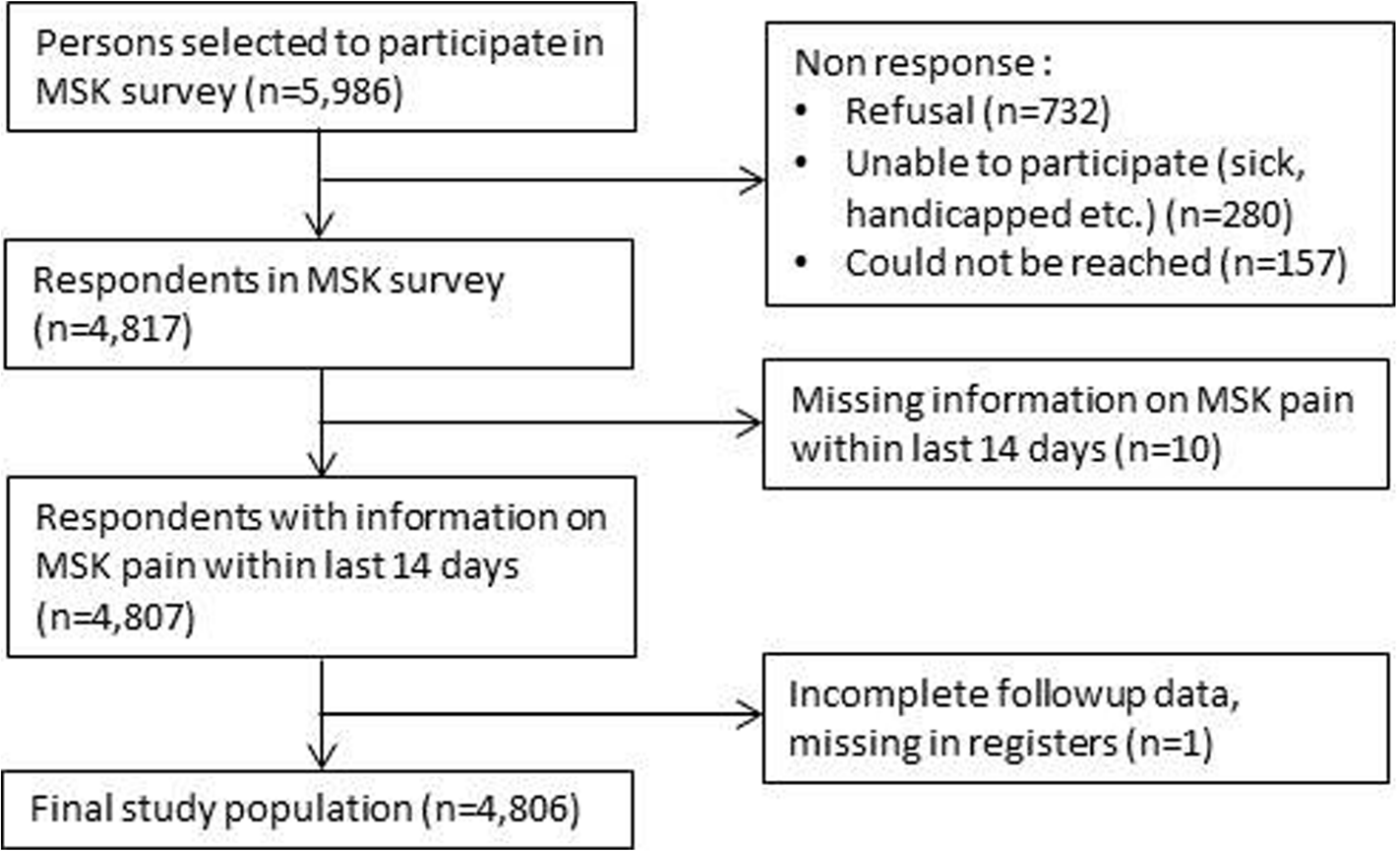
Flow-chart of participants through study

A total of 4817 persons (80.5%) of the 5986 invited persons responded to the questionnaire. Reasons for non-response among non-responders (*n* = 1169): refusal (*n* = 732), unable to participate (sick, handicapped, etc.) (*n* = 280), could not be reached (*n* = 157). Moreover, 10 persons had missing information on MSK pain, and one had incomplete follow-up data. Therefore, 4806 persons, corresponding to 80.3% of the initial population, were included in analyses (Fig. 1).

Baseline characteristics of all respondents showed that 41% of the respondents had experienced MSK pain within the last 14 days (24.0% in one area, 12.7% in two areas and 4.3% widespread pain) (Table 1). Examining the pain intensity showed that 60.1% had no pain, 24.9% mild pain, 11.7% moderate pain and 3.2% strong pain (Table 1). Among those with strong pain, 29% also had widespread pain (data not shown).

**Table 1:** General characteristics of the study population at baseline

Variables	All (***n*** = 4806)	MSK pain within last 14 days ***n*** = 1971 (41.0%)	No MSK pain ***n*** = 2835 (59.0%)
***Age (mean ± SD)***	44.5 ± 0.27	46.9 ± 0.42	42.9 ± 0.34
***Age***			
**16–44 (%)**	54.7	37.3	62.7
**45–64 (%)**	27.6	43.6	56.4
**≥ 65**	17.8	48.6	51.4
***Sex***			
**Men (%)**	47.9	42.3	51.7
**Women (%)**	52.2	57.7	48.3
***Marital status***			
**Married or cohabitant (%)**	66.9	66.7	67.1
**Widow(er) (%)**	7.8	9.5	6.6
**Divorced (%)**	5.9	6.9	5.2
**Unmarried (%)**	19.4	16.8	21.1
***Education level (years)***			
**< 10 years (%)**	28.6	30.9	26.9
**10 years (%)**	9.8	8.6	10.6
**11–12 years (%)**	10.4	9.6	10.9
**13–14 years (%)**	26.7	26.0	27.5
**15+ years (%)**	9.25	8.0	10.2
**Missing (%)**	15.2	16.9	13.9
***Physical activity***			
**Vigorous or Moderate (%)**	21.1	17.9	23.4
**Light (%)**	65.5	65.1	65.7
**Sedentary (%)**	13.4	17.0	10.9
***Stressed in daily life***			
**Often (%)**	6.0	8.3	4.4
**From time to time (%)**	30.5	31.9	29.6
**Never or almost never (%)**	63.5	59.5	65.7
**Don’t know (%)**	0.3	0.3	0.4
***Meet up with friends or acquaintance***			
**Daily or almost daily (%)**	29.3	28.2	30.1
**1–2 times a week/month (%)**	54.5	63.6	65.1
**Less often or never (%)**	6.2	8.2	4.8
***Comorbidity***^***a***^			
**No (%)**	87.8	84.9	89.8
**Low (%)**	8.2	10.7	6.6
**Medium/high (%)**	4.0	4.5	3.6
***Smoking***^***b***^			
**Daily (%)**	41.9	44.7	40.0
**Occasional (%)**	3.6	3.4	3.7
**No (%)**	54.5	51.9	56.3
**Spread of pain**			
**No areas (%)**	59.0		
**One area (%)**	24.0		
**Two areas (%)**	12.7		
**Three areas (widespread) (%)**	4.3		
**Pain intensity categorical**			
**No pain (0–4) (%)**	60.1		
**Mild (5–44) (%)**	24.9		
**Moderate (45–74) (%)**	11.7		
**Strong (75–100) (%)**	3.2		

^a^No comorbidity: Charlson score = 0; low comorbidity: Charlson score = 1; and medium/high: Charlson score = ≥2

^b^*N* = 3198: question on smoking only applied in two out of three interview session

The mean age was 44.5 ± 0.27 years, persons with MSK pain within the last 14 days being slightly older than respondents without MSK pain (46.9 vs. 42.9 years, *p* < 0.001). A higher proportion of respondents with MSK pain had comorbidity, a sedentary lifestyle, low educational level (< 10 years), less frequent or no contact with friends, were often stressed in daily life, and were daily smokers compared to persons without MSK pain (Table 1).

Complete follow-up information was available for practically all respondents (Fig. 1); however, information on cause of death was missing for 77 persons due to a delay in registering (information was unavailable in RCD for the year of 2013) or because persons had died aboard. Mean follow-up time in the total cohort was 19.1 years [range 0.04–23.3 years], giving a total of 91,868 person-years. A total of 201 persons emigrated and 1372 persons died during follow-up. The largest proportion died of cardiovascular disease (32.2%) followed by cancer (25.8%) (Table 2).

**Table 2:** Causes of Death (*n* = 1372)

Cause of Death	N (%)
Cardiovascular diseases	443 (32.3)
Cancer	354 (25.8)
Respiratory diseases	128 (9.3)
Psychological diseases (incl. Alzheimer’s/dementia)	55 (4.0)
Gastrointestinal diseases	63 (4.6)
Other	203 (14.8)
Missing (no information on death cause due to delay in registration, or death aboard)	77 (5.6)

**Fig. 2:**
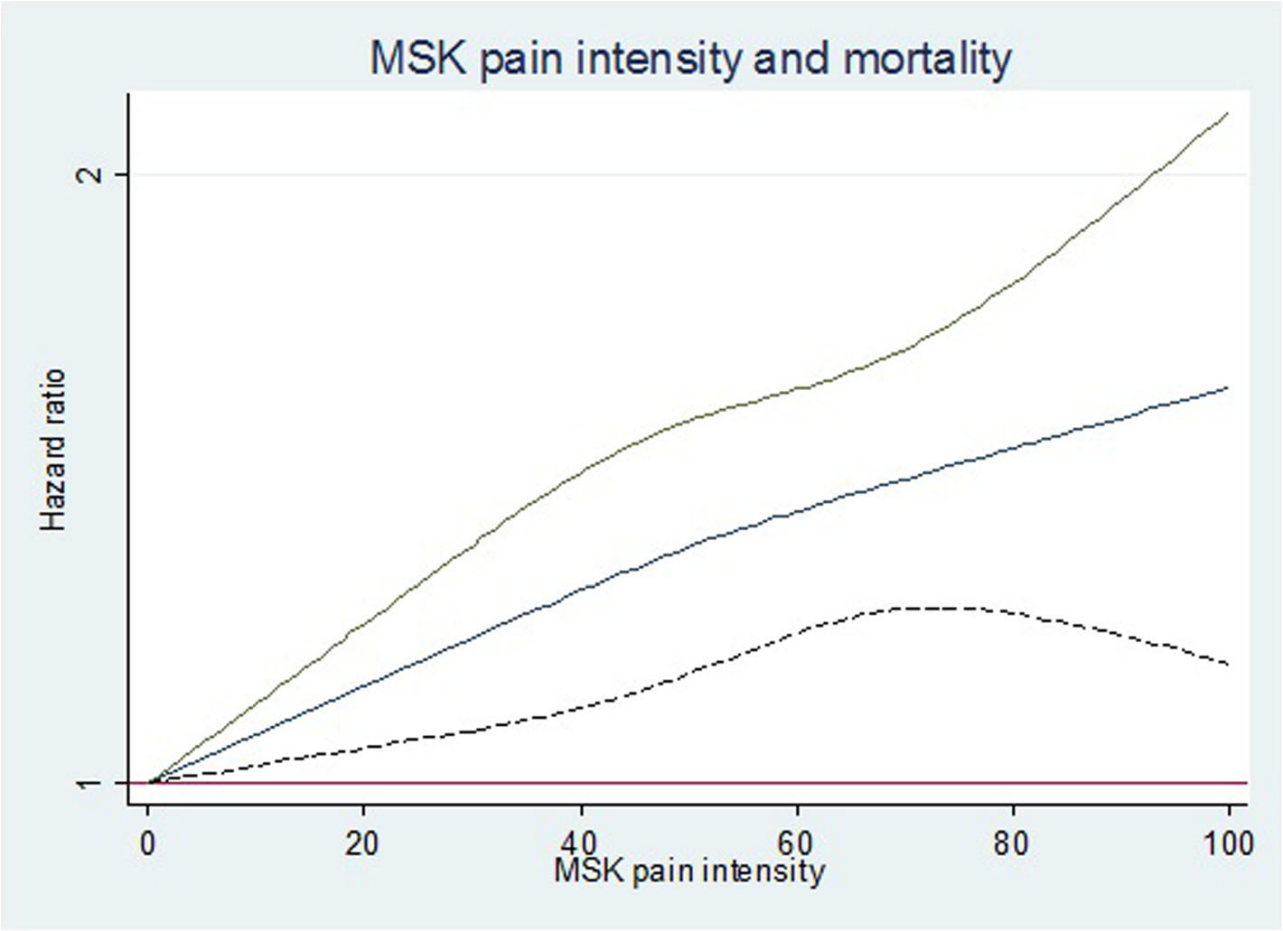
MSK pain intensity and all-cause mortality when applying cubic splines

All-cause mortality rates increased for both men and women with severity of MSK pain within last 14 days (Table 3). The highest rates were observed in persons with widespread pain (men: 36.2 per 1000 person-years; women: 34.9 per 1000 person-years). The age-adjusted Kaplan-Meier curves for all-cause mortality are shown in Figures 1, 2, 3 in supplemental material.

**Table 3:**
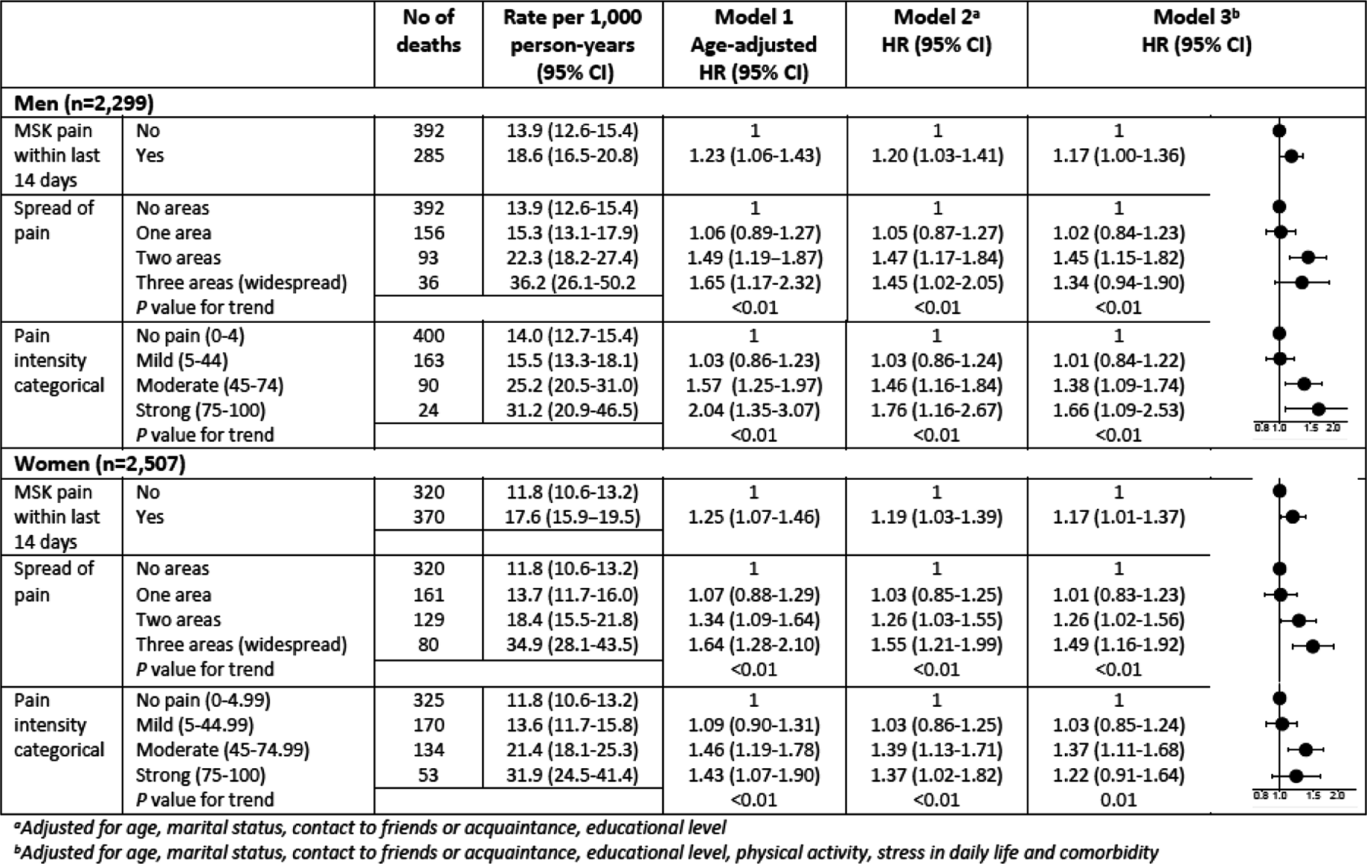
All-cause mortality rates per 1000 person-years and hazard ratios (HR) for men and women in relation to MSK pain within last 14 days (1372 deaths)

The analyses of all-cause mortality showed the same tendencies of increasing mortality with higher spread and intensity of pain. MSK pain within last 14 days yielded *c*-statistics of 0.544 and 0.887 with age added, whereas spread of pain reached *c*-statistics of 0.557 (0.872 including age) and intensity of pain was 0.556 (0.872 including age).

The fully adjusted Cox regression model (model 3) showed a tendency of increased risk for all-cause mortality among men with MSK pain within last 14 days compared to men without MSK pain (HR = 1.17, 95% CI: 1.00–1.36; *P* = 0.053). Moreover, men with MSK pain in two areas had an increased risk of all-cause mortality compared to men without MSK pain (HR = 1.45, 95% CI: 1.15–1.82). Similarly, men with moderate or strong pain have a higher risk of mortality compared to men without MSK pain (HR = 1.38, 95% CI: 1.09–1.74 and HR = 1.66, 95% CI: 1.09–2.53, respectively) (Table 3).

Among women, the fully adjusted Cox regression showed HR of 1.17 for all-cause mortality among those with MSK pain within last 14 days compared to women without MSK pain (95% CI: 1.01–1.37). Women with MSK pain in two or three areas had an increased risk of all-cause mortality compared to women without MSK pain (HR = 1.26, 95% CI: 1.02–1.56 and HR = 1.49, 95% CI: 1.16–1.92, respectively). Further, women with moderate pain had a 37% higher risk of all-cause mortality compared to women without MSK pain (95% CI: 1.11–1.68) (Table 3).

To explore the potential confounding effect of smoking, we performed sensitivity analyses among the subgroup who answered questions on smoking behaviour (*N* = 3173). These analyses showed the same tendencies with almost similar HRs as in analyses adjusted for age and other confounders. This applied for both men and women (data are shown in Table 1 in the supplemental material).

None of the multiplicative effect modification analyses between MSK pain (yes/no, spread of pain, pain intensity) and education as well as MSK pain and comorbidity were statistically significant. Sensitivity analyses delaying start of follow-up with 2 years showed the same results as the main analyses (data not shown).

When examining the different causes of mortality, persons with MSK pain within last 14 days generally had higher cause-specific mortality rates compared to persons without MSK pain; especially persons with widespread or strong pain. Moreover, there was a tendency of increased cardiovascular mortality in persons with strong MSK pain compared to persons without pain (HR = 1.49, 95% CI: 1.01–2.21). In analyses on cancer mortality, MSK pain in two areas was associated with cancer mortality (HR = 1.58, 95% CI: 1.18–2.11). The same was seen for persons with moderate pain who had an increased cancer mortality compared to persons without MSK pain (HR = 1.56, 95% CI: 1.16–2.09). Moreover, persons with widespread pain had a more-than-double respiratory mortality risk compared to persons without MSK pain (HR = 2.43, 95% CI: 1.42–4.17). Finally, higher gastrointestinal mortality among persons with moderate pain compared to persons with no pain was observed (HR = 1.94, 95% CI: 1.03–3.63) (Table 4).

**Table 4:**
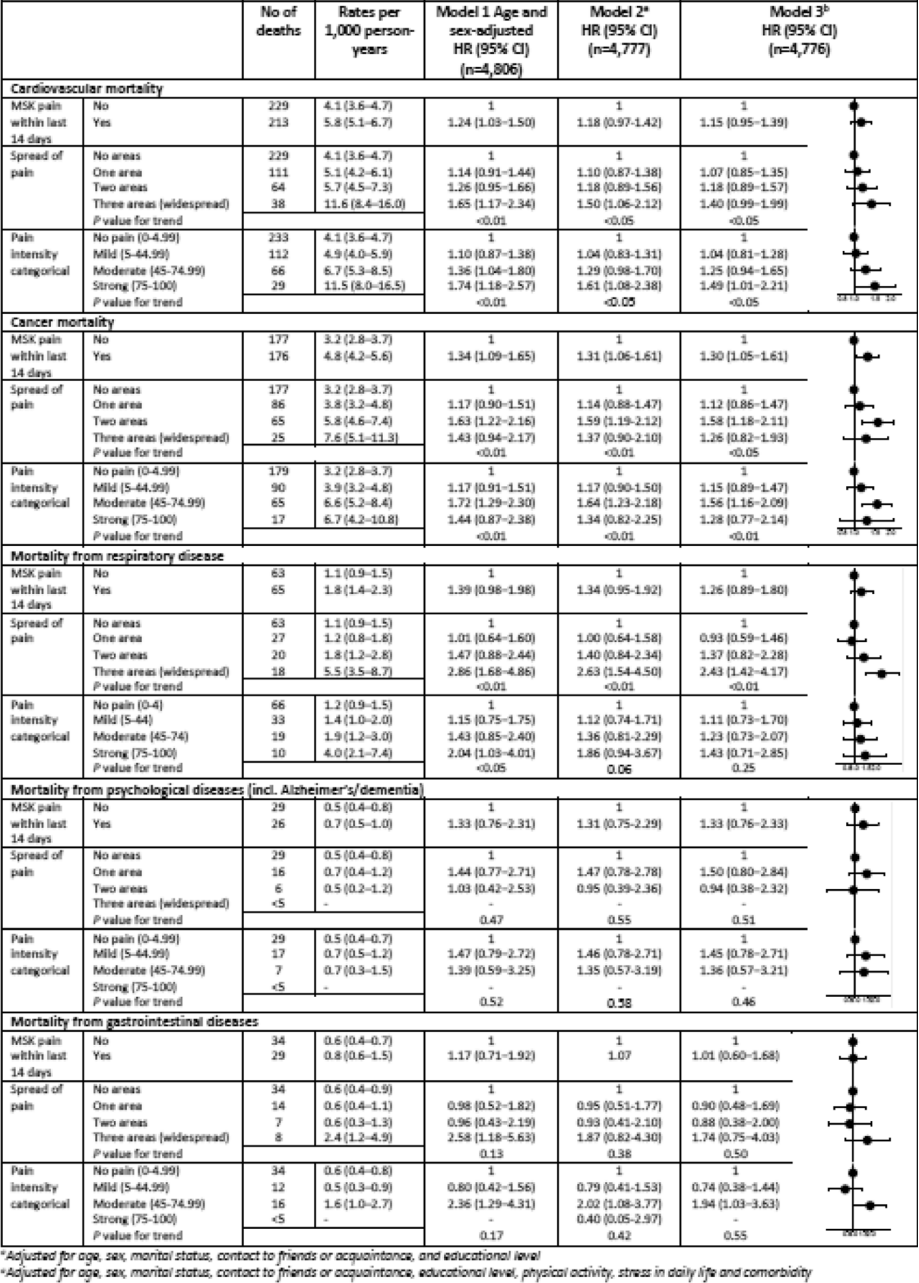
Cause-specific mortality per 1000 person-years and hazard ratios in relation to MSK pain within last 14 days

The relationship between MSK pain intensity and all-cause mortality was not linear (data not shown); therefore, we investigated the relationship using cubic splines. Figure 2 shows hazard ratio for all-cause mortality in relation to MSK pain intensity when applying cubic splines. As seen, there is a tendency of increased risk of all-cause mortality with increasing MSK pain intensity.

## Discussion

The results from this prospective population-based study showed higher all-cause and cause-specific mortality rates among persons experiencing MSK pain within the last 14 days. Moreover, increasing risks of all-cause mortality with increasing spread and intensity of MSK pain was observed―also when adjusting for a range of potential confounders. A high risk of all-cause mortality was particularly seen among men with strong pain and among women with widespread pain compared to men and women without MSK pain.

However, the discriminatory accuracy of MSK pain was low. MSK pain within last 14 days yielded c-statistics of 0.544, whereas spread of pain was 0.557 and intensity of pain was 0.556. This indicates that this information cannot stand alone if used for predicting mortality risk – the discriminatory accuracy increased substantial when including age (c-statistics of 0.872 to 0.887). Therefore, any attempt of intervention based on the existence of MSK pain alone to prevent mortality will be inefficient; other risk factors (e.g. age) must also be considered.

Diverse approaches to define musculoskeletal pain are often found in studies of the association between MSK pain and mortality, which influences the prevalence of MSK pain. Some studies include chronic pain (in some instances defined as fibromyalgia, using the classification of the American College of Rheumatology (ARC) [11]), pain spread (regional or widespread (defined as pain in three, four or more regions or sites [18]), while others have looked at the specific location of MSK pain e.g. back pain [34]. In the present study, we focused on different aspects of MSK pain within a 14-day period, which distinguishes our approach from that used in most other published studies. However, this approach was used as we wanted to investigate different levels of experienced MSK pain and include all persons who have experienced some kind of MSK pain (not only chronic pain). Moreover, we used the following definition of widespread pain: pain in three different regions 1) upper body (elbows, shoulders, hands), 2) lower body (hips, knees, feet/ankles), and 3) trunk (neck, chest, upper- and lower back), which was inspired by Overland and colleagues [25]. It is important to note that this is a modified version of the ARC 1990 definition of widespread pain, where pain is considered widespread when all of the following are present: pain in the left side of the body, pain in the right side of the body, pain above the waist, and pain below the waist) [24].

The number of included covariates in previous studies also differed to a great extent. The majority of studies only adjusted for age (and maybe sex), and our main results could be compared with these, showing an increased risk of all-cause mortality in persons with pain [10, 11, 13, 17, 29, 34, 35]. However, we also included other covariates such as comorbidity and stress, and even though some of the observed associations varied when adjusting for additional confounders, overall the same tendencies in effect estimates were seen. In other studies, the inclusion of other covariates revealed more inconsistent results [17–19], e.g. in one study the authors concluded that adjustment for lifestyle factors eliminated the excess risk of all-cause mortality [17].

We are not aware of any studies using VAS to investigate the intensity of MSK pain in relation to all-cause mortality. Interestingly, the group of persons with strong pain and the group of persons with widespread MSK pain was only partly overlapping, as it was shown that only 29% of persons with strong pain also had widespread pain. In comparing the findings related to MSK pain intensity, Torrance et al. measured the severity of pain with the Chronic Pain Questionnaire and found that survival among those reporting severe chronic pain was significantly worse than among those reporting mild or no chronic pain, irrespective of the cause of pain [19].

The reasons for the relationship between pain and increased risk of mortality remain unsolved; is the increased mortality a result of MSK pain, or is it an effect of other causes (or perhaps an interaction of different causes)? Is it the pain itself or something about the pain experience that has biological significance for mortality? We tested for interactions in relation to education and comorbidity without revealing any associations, but other complex interactions could be present. A recently published Danish study assessed the mortality risk among opioid users with chronic pain and found that all-cause mortality was highest among long-term opioid users [35]. In the present study, we did not investigate associations with opioid or other drugs that might explain part of the relationship. Another explanation could be that MSK pain in fact is caused by other diseases. However, by taking comorbidity into account and performing sensitivity analyses delaying start of follow-up by 2 years, we tried to exclude his potential explanation. In continuation hereof, Jordan and Croft compared mortality rates among patients with MSK problems (pain) with those of patients without such problems, and concluded that the excess mortality was only partly explained by cancer and other comorbidity [34]. In general, more studies are needed to determine the nature of the association.

When looking at the risk of cause-specific mortality, the findings of others have been conflicting. We found an increased risk of cardiovascular mortality in age- and sex-adjusted analyses among persons experiencing MSK pain. Others also showed increased risk among persons with pain when adjusted for age and sex [17, 19, 29], whereas others again have found no association [11, 35]. Likewise, our analyses showed a tendency of increased risk of cancer mortality among persons with MSK pain. McBeth et al. found an 80% higher risk of cancer mortality among persons with widespread pain [29], while Macfarlane et al. discovered an approx. Twice as high risk of cancer mortality in persons with widespread pain compared to persons with no pain [11]. In contrast, other studies showed no association [17, 18]. Lastly, we found an increased risk of mortality from respiratory diseases among persons with widespread pain. Except from one study [19], this differs from what most others have found [11, 18, 29]. In general, none of the analyses of the cause-specific mortality have excluded persons with the given disease corresponding to the cause of death at baseline; further studies have to take this into account to fully understand the associations.

Our study has several important strengths. First, it was based on a prospective population-based design with long follow-up, comprising both sexes and covering a broad age range. The data collection was interview-based, and a relatively high response percentage was obtained (80.5%), even among the oldest (e.g. 75.1% among the 70–79–years-old). The linkage to national registers ensured complete follow-up. The registers are considered to be internationally the most comprehensive with high validity. Any misclassification of the cause of death is considered to be non-differential as the register data were collected independently of our research―and, if any, would bias the associations towards the null. Furthermore, the study included different aspects of MSK pain, and analyses included other factors e.g. comorbidity that could affect the relationship between MSK pain and mortality. Moreover, we used a robust method (Cox regression), taking person-years and censuring into account. However, it is important to note that when applying Cox regressions for analysing the cause-specific mortality it is a limitation that it doesn’t take competing risk from other causes into account.

Another limitation is that we had no information on body mass index (BMI), which is a known and important risk factor for MSK pain [7, 36]. Likewise, BMI (body mass index) is an established risk factor of e.g. cardiovascular diseases, cancer and mortality. Unfortunately, BMI was not included in the survey. Moreover, the possibility of other unmeasured confounders such as e.g. sleep problems cannot be excluded. Furthermore, we only had information on smoking for a subgroup of the population, which precluded the inclusion of this variable in the analyses using the whole study population. A supplemental analysis revealed that the two populations were similar in terms of age, sex, level of education, number of deaths and the proportion that have experienced MSK pain within the last 14 days (data not shown). Moreover, sensitivity analyses showed comparable HRs. In a study by Macfarlane et al., the authors similarly found no differences in the risk of cancer mortality when including smoking status in their analysis [11].

Even though the response rate was high; we cannot exclude the possibility of selection bias. We therefore compared the respondents with the non-respondents at baseline. As is often the case with surveys, respondents tended to be younger, have a higher education and married (data not shown). Nevertheless, the observed associations between MSK pain and mortality are still believed to reflect those in the background population, but it is the size of the risk estimate that could be affected. As the questionnaire had a specific focus on musculoskeletal conditions, we cannot rule out the possibility that people with musculoskeletal problems to a greater extent have answered the questionnaire.

Another potential limitation is that data were collected in 1990–1991 and patterns of MSK pain may have changed in the population since then. Further, in present study we used the respondent’s subjective evaluation of pain. It would have been preferable if we had been able to include clinical judgement on this matter. However, we would argue that the risk of misunderstanding (and misclassification) of the exposure was minimized by the interview-based data collection method, where the interviewer shows a diagram with the different body areas clearly marked. Moreover, the 2-week recall period used may have decreased information bias, because in many cases longer recall periods result in misclassification and hence information bias [37]. The reliability of the question on MSK pain was not evaluated in present study.

Moreover, in the present study comorbidity and the calculation of the Charlson comorbidity index was based on register information on hospitalisation until baseline, which yields a possibility of confounding by other prevalent comorbidity not severe enough to have led to hospitalisation.

A final limitation in analyses is the sample size and numbers of deaths, which for some of the death causes resulted in strata with small numbers of cases, and therefore the most reliable results are for analyses of all-cause mortality as well as cancer and cardiovascular mortality. This may explain why we did not find statistically significant HR for less common causes of death; however, these findings should be studied further. Likewise, a power issue could also apply to the different analyses of effect modifications.

## Conclusion

In summary, the results of this study indicate that persons experiencing MSK pain within the last 14 days at baseline have higher mortality- and cause-specific mortality rates. In both women and men, a trend of increasing risk of all-cause mortality with increasing spread and intensity of MSK pain was observed. The results confirm previous findings regarding the relationship between pain and mortality and provide new insights into the long-term consequences of MSK pain; in particular it seems that those experiencing strong or widespread pain are especially vulnerable. However, when evaluating these results, it is important to take the low discriminatory accuracy and the possibility of unmeasured confounders into account (we had no information on e.g. BMI etc).

## Supplementary information

**Additional file 1 Figure S1.** Kaplan-Meier survival curve MSK pain within last 14 days (yes; no).

**Additional file 2 Figure S2.** Kaplan-Meier survival curve spread of pain (no areas; one area; two areas; three areas (widespread)).

**Additional file 3 Figure S3.** Kaplan-Meier survival curve pain intensity categorical (no pain (0–4); mild (5–44); moderate (45–74); strong (75–100)).

**Additional file 4 Table S1.** All-cause mortality rates per 1000 person-years and hazard ratios (HR) for men and women in relation to MSK pain within last 14 days (894 deaths) among participants with information on smoking.

## Data Availability

The data that support the findings of this study are available from Danish National Institute of Public Health and Statistic Denmark, but restrictions apply. These data were used under licence for the current study, and are not publicly available. The data are, however, available from the authors upon reasonable request and with permission of Statistic Denmark.

## Abbreviations

MSK: Musculoskeletal
YLD: Years lived with disability
VAS: Visual analog scale
RCD: Danish Register of Causes of Death
ICD: International Classification of Diseases
CRS: Civil Registration System
ISCED: International Standard Classification of Education
SE: Standard error
HR: Hazard ratio
95 % CI: 95 % confidence intervals
ARC: American College of Rheumatology
BMI: Body mass index
