# Supplementary figures and images for: Mortality among persons experiencing musculoskeletal pain: a prospective study among Danish men and women
Source: BMC Musculoskelet Disord. 2020 Oct 8;21:666. doi: 10.1186/s12891-020-03620-8 (PMC7545937; doi:10.1186/s12891-020-03620-8)

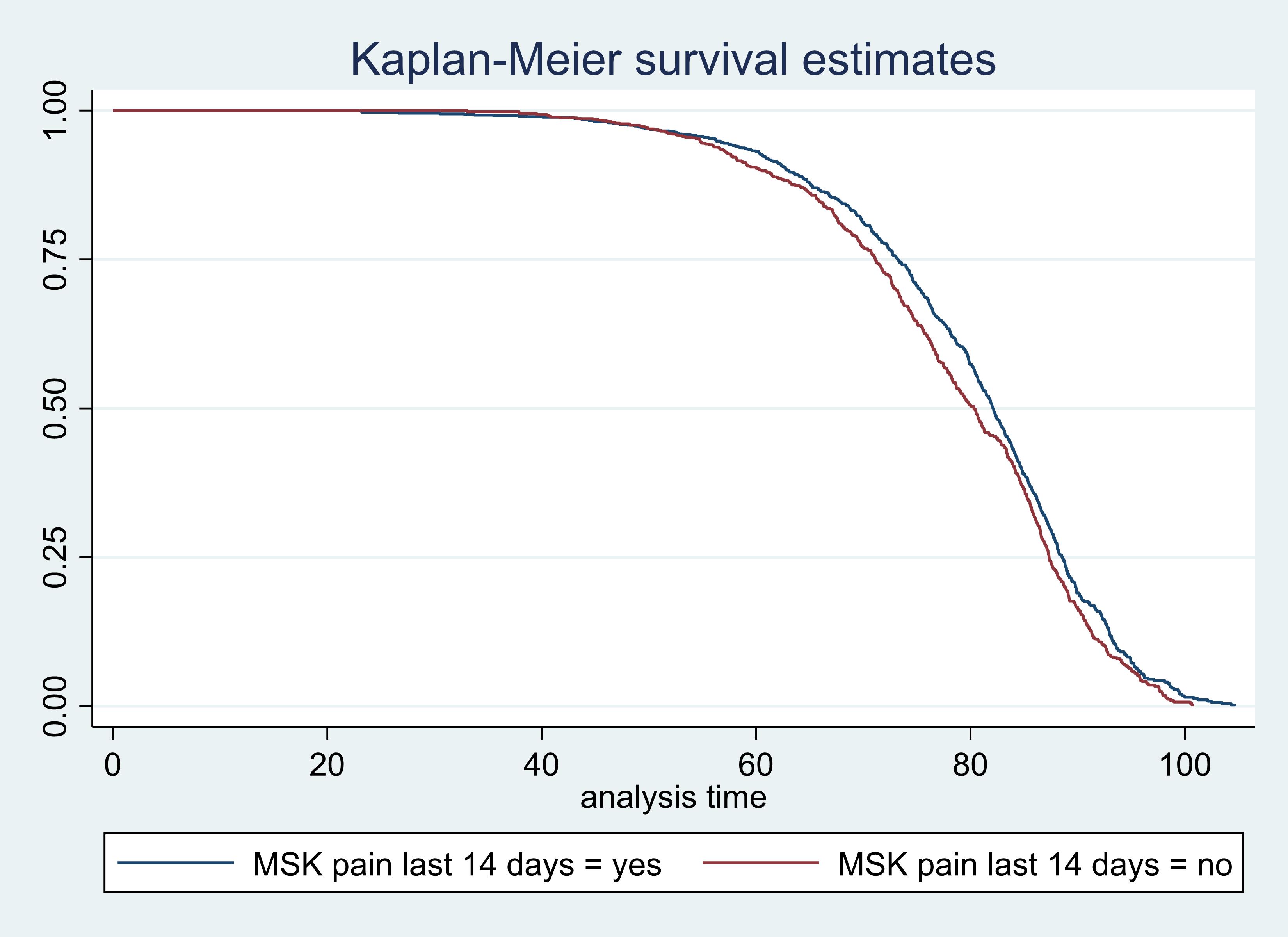

Supplement: Supplementary file 1 — Additional file 1 Figure S1. Kaplan-Meier survival curve MSK pain within last 14 days (yes; no). [file 12891_2020_3620_MOESM1_ESM.jpg]

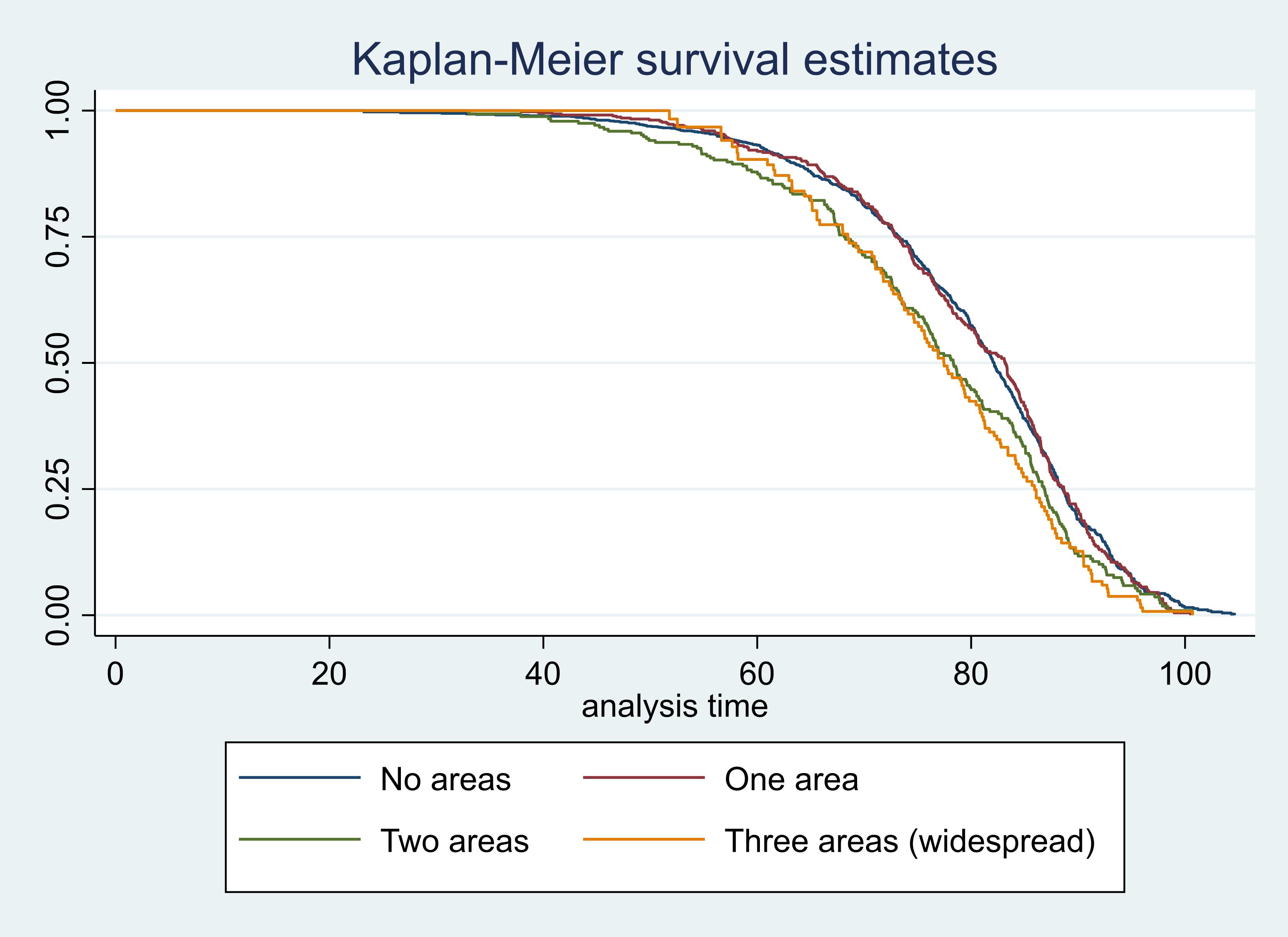

Supplement: Supplementary file 2 — Additional file 2 Figure S2. Kaplan-Meier survival curve spread of pain (no areas; one area; two areas; three areas (widespread)). [file 12891_2020_3620_MOESM2_ESM.jpg]

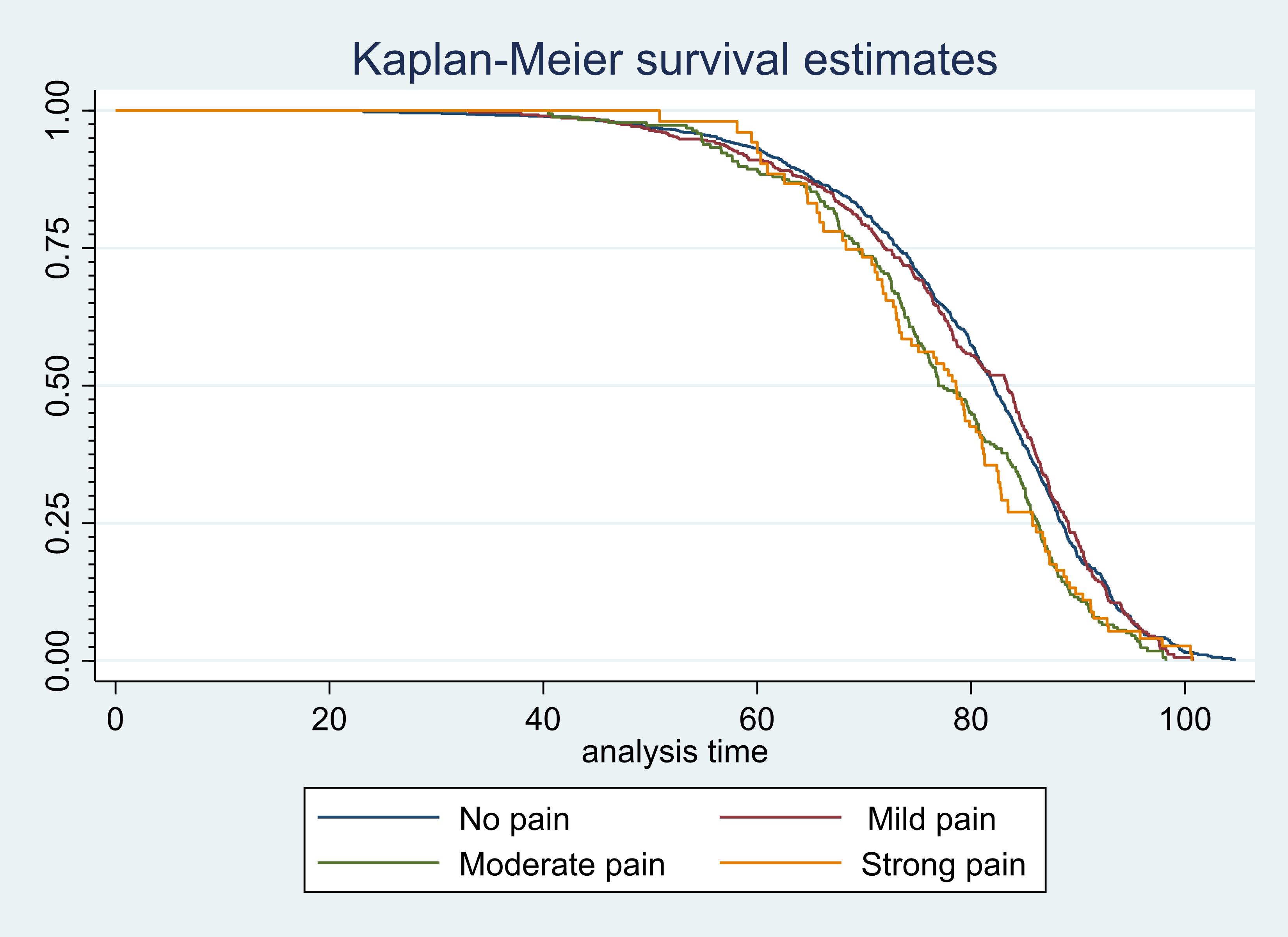

Supplement: Supplementary file 3 — Additional file 3 Figure S3. Kaplan-Meier survival curve pain intensity categorical (no pain (0–4); mild (5–44); moderate (45–74); strong (75–100)). [file 12891_2020_3620_MOESM3_ESM.jpg]
