# Supplementary material for: Mortality among persons experiencing musculoskeletal pain: a prospective study among Danish men and women
Source: BMC Musculoskelet Disord. 2020 Oct 8;21:666. doi: 10.1186/s12891-020-03620-8 (PMC7545937; doi:10.1186/s12891-020-03620-8)
Supplement: Supplementary file 4 — Additional file 4 Table S1. All-cause mortality rates per 1000 person-years and hazard ratios (HR) for men and women in relation to MSK pain within last 14 days (894 deaths) among participants with information on smoking. [file 12891_2020_3620_MOESM4_ESM.docx]

| **Supplemental table 1. All-cause mortality rates per 1,000 person-years and hazard ratios (HR) for men and women in relation to MSK pain within last 14 days (894 deaths) among participants with information on smoking** | | | | |
| --- | --- | --- | --- | --- |
|  | | **No of deaths** | **Rates per 1,000 person-years** | **Adjusted^+^**  **HR (95% CI)** |
| **Men (n=1,541)** | |  | |  |
| MSK pain within last 14 days | No  Yes | 257  180 | 13.6 (12.0−15.3)  17.3 (14.9−20.0) | 1  1.12 (0.92−1.37) |
| Spread of pain | No areas  One area  Two areas  Three areas (widespread)  *P* value for trend | 257  95  58  27 | 13.6 (12.0−15.3)  14.0 (11.5−17.2)   - 1. (15.5−26.0)   35.4 (24.3−52.0) | 1  0.99 (0.78−1.27)  1.26 (0.94−1.67)  1.45 (0.97−2.19)  <0.05 |
|  |  |  | |  |
| Pain intensity categorical | No pain (0-4.99)  Mild (5-44.99)  Moderate (45-74.99)  Strong (75-100)  *P* value for trend | 263  106  54  14 | 13.6 (12.1−15.3)  14.9 (12.3−18.0)  22.3 (17.0−29.1)  30.5 (18.6−51.5) | 1  0.97 (0.76−1.22)  1.43 (1.06−1.94)  1.52 (0.87−2.65)  <0.05 |
|  |  |  | |  |
| **Women (n=1,653)** | | | | |
| MSK pain within last 14 days | No  Yes | 207  250 | 11.7 (10.2−13.4)  18.3 (16.1−20.7) | 1  1.11 (0.91−1.34) |
| Spread of pain | No areas  One area  Two areas  Three areas (widespread)  *P* value for trend | 207  112  79  59 | 11.7 (10.2−13.4)  15,6 (13.0−18.8)   - 1. (13.1−20.4)   35.4 (27.5−45.7) | 1  0.98 (0.78−1.25)  1.15 (0.88−1.51)  1.39 (1.03−1.88)  <0.05 |
|  |  |  | |  |
| Pain intensity categorical | No pain (0-4.99)  Mild (5-44.99)  Moderate (45-74.99)  Strong (75-100)  *P* value for trend | 210  117  93  32 | 11.6 (10.2−13.3)  14.7 (12.3−17.6)   - 1. (17.9−26.8)   28.9 (20.5−40.9) | 1  1.03 (0.82−1.30)  1.13 (0.87−1.45)  1.29 (0.87−1.91)  0.17 |
|  |  |  | |  |

*^+^ adjusted for age, marital status, contact to friends or acquaintance, physical activity, stress in daily life, educational level, comorbidity and smoking*
